# Substantia Nigra Integrity Correlates with Sequential Working Memory in Parkinson's Disease

**DOI:** 10.1523/JNEUROSCI.0242-21.2021

**Published:** 2021-07-21

**Authors:** Wenyue Liu, Changpeng Wang, Tingting He, Minghong Su, Yuan Lu, Guanyu Zhang, Thomas F. Münte, Lirong Jin, Zheng Ye

**Affiliations:** ^1^Institute of Neuroscience, Key Laboratory of Primate Neurobiology, Center for Excellence in Brain Science and Intelligence Technology, Chinese Academy of Sciences, Shanghai 200031, China; ^2^University of Chinese Academy of Sciences, Beijing 100049, China; ^3^Department of Neurology, Zhongshan Hospital, Fudan University, Shanghai 200032, China; ^4^Institute of Psychology, Chinese Academy of Sciences, Beijing 100101, China; ^5^Department of Neurology, University of Lübeck, 23538 Lübeck, Germany; ^6^Shanghai Center for Brain Science and Brain-Inspired Intelligence Technology, Shanghai, 201210, China

**Keywords:** basal ganglia, functional MRI, neuromelanin-sensitive MRI, Parkinson's disease, sequential working memory, substantia nigra

## Abstract

Maintaining and manipulating sequences online is essential for daily activities such as scheduling a day. In Parkinson's disease (PD), sequential working memory deficits have been associated with altered regional activation and functional connectivity in the basal ganglia. This study demonstrates that the substantia nigra (SN) integrity correlated with basal ganglia function and sequencing performance in 29 patients with PD (17 women) and 29 healthy controls (HCs; 18 women). In neuromelanin-sensitive structural magnetic resonance imaging (MRI), PD patients showed smaller SNs than HCs. In a digit-ordering task with functional MRI (fMRI), participants either recalled sequential digits in the original order (pure recall) or rearranged the digits and recalled the new sequence (reorder and recall). PD patients performed less accurately than HCs, accompanied by the caudate and pallidal hypoactivation, subthalamic hyperactivation, and weakened functional connectivity between the bilateral SN and all three basal ganglia regions. PD patients with larger SNs tended to exhibit smaller ordering-related accuracy costs (reorder and recall vs pure recall). This effect was fully mediated by the ordering-related caudate activation. Unlike HCs, the ordering-related accuracy cost correlated with the ordering-related caudate activation but not subthalamic activation in PD patients. Moreover, the ordering-related caudate activation correlated with the SN area but not with the daily dose of D_2/3_ receptor agonists. In PD patients, the daily dose of D_2/3_ receptor agonists correlated with the ordering-related subthalamic activation, which was not related to the accuracy cost. The findings suggest that damage to the SN may lead to sequential working memory deficits in PD patients, mediated by basal ganglia dysfunction.

**SIGNIFICANCE STATEMENT** We demonstrate that damage to the SN correlates with basal ganglia dysfunction and poor sequencing performance in PD patients. In neuromelanin-sensitive MRI, PD patients showed smaller SNs than healthy controls. In a digit-ordering task with fMRI, PD patients' lower task accuracy was accompanied by the caudate and pallidal hypoactivation, subthalamic hyperactivation, and weakened functional connectivity between the SN and basal ganglia. PD patients with larger SNs exhibited greater ordering-related caudate activation and lower ordering-related accuracy cost when sequencing digits. PD patients with more daily exposure to D_2/3_ receptor agonists exhibited greater ordering-related subthalamic activation, which did not reduce accuracy cost. It suggests that the SN may affect sequencing performance by regulating the task-dependent caudate activation in PD patients.

## Introduction

The ability to maintain and manipulate sequential information online is essential for a broad spectrum of daily activities (e.g., scheduling a day). In Parkinson's disease (PD), sequential working memory deficits can lead to difficulties in sorting words and numbers (Cooper et al., 1991; Gabrieli et al., 1996), organizing sequential steps to achieve goals (Owen, 1997; West et al., 1998; Sullivan et al., 2009), and understanding the relationship among events that are not stated in the order they occurred (Natsopoulos et al., 1991; Al-Khaled et al., 2012). We have linked poor sequencing performance in PD with altered regional activation and functional connectivity in the basal ganglia (Ye et al., 2021). In this study, we further investigate whether damage to the substantia nigra (SN) could contribute to the basal ganglia's functional changes using neuromelanin-sensitive structural as well as functional magnetic resonance imaging (fMRI).

Recently we described a neural system for sequential working memory, comprising the lateral prefrontal cortex, posterior parietal cortex, caudate nucleus, globus pallidus, subthalamic nucleus, and thalamus (Ye et al., 2020). Both PD and normal aging can compromise this neural system but in different manners. The age effect led to prefrontal and parietal hyperactivation and a weakened psychophysiological interaction between the prefrontal/parietal regions and the supplementary motor area (Ye et al., 2020). In contrast, the disease effect was manifested as subthalamic and pallidal hyperactivation and weakened functional connectivity between the subthalamic nucleus and striatum (Ye et al., 2021).

Cognitive decline in early PD may correlate with the spread of misfolded α-synuclein (Braak et al., 2006), which hits the locus ceruleus (LC) in stage 2 and the SN in stage 3 of the disease. We hypothesize that the basal ganglia's functional changes during sequential working memory correlate with the degree of SN integrity. To test this hypothesis, we measured the SN integrity *in vivo* using neuromelanin-sensitive MRI. Neuromelanin is a by-product of catecholamine synthesis, existing in SN dopamine neurons and LC noradrenaline neurons (Zecca et al., 2001; Fedorow et al., 2005). In PD, MRI signals of the neuromelanin are remarkably diminished in the SN and LC (Sasaki et al., 2006; Martin-Bastida et al., 2017; Wang et al., 2018), consistent with the loss of dopamine and noradrenaline neurons in these nuclei (Isaias et al., 2016; Ito et al., 2017). It raises the question of whether changes in SN integrity might have an impact on neural processes of sequential working memory.

**Figure 1.**
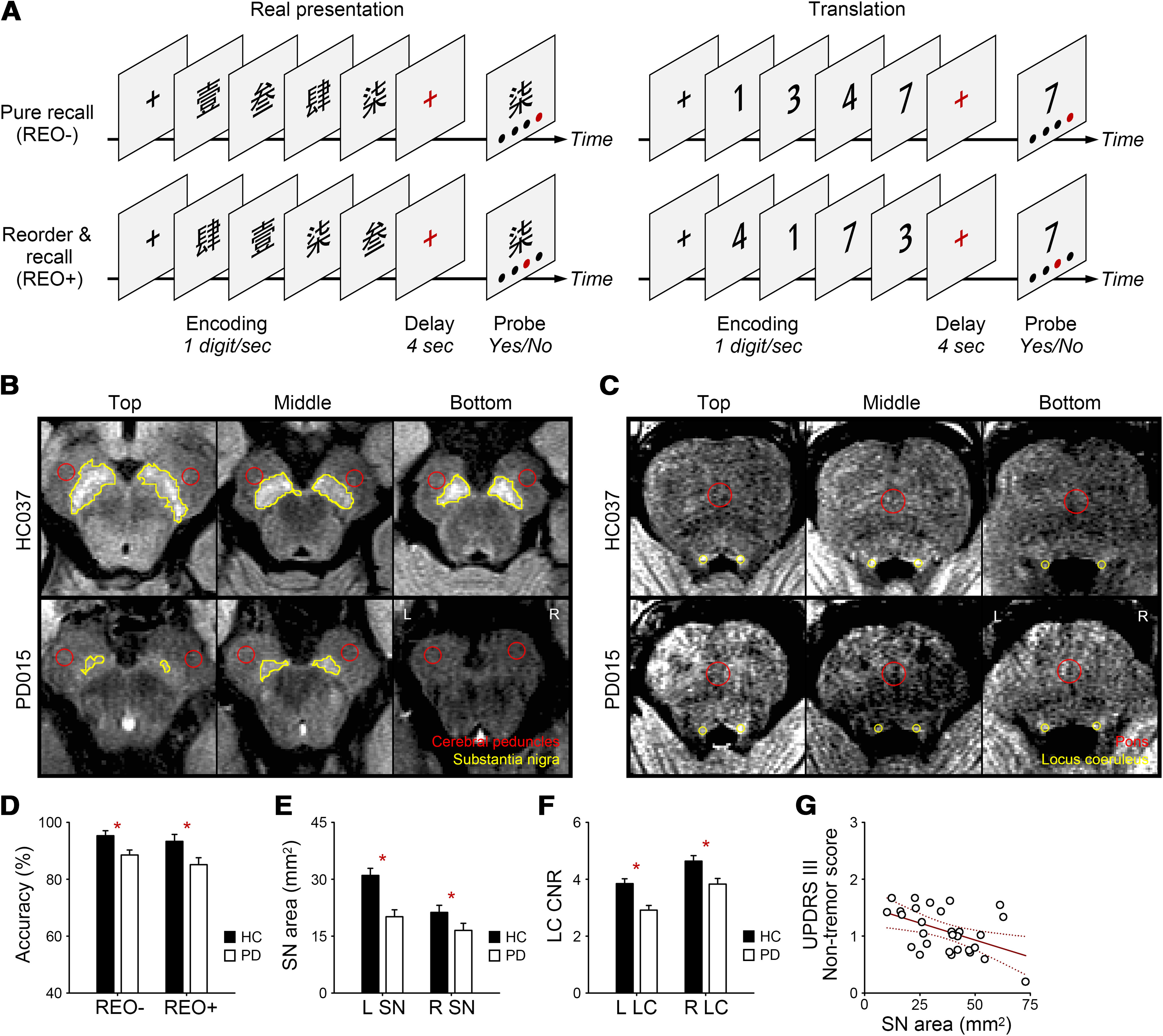
Digit ordering task, behavioral data, and neuromelanin signals. ***A***, The digit-ordering task includes interleaved pure recall (REO−) and reorder and recall trials (REO+). ***B***, In neuromelanin-sensitive MRI, the SN was identified as a cluster of voxels with a signal intensity of 2.5 SDs above the mean signal intensity of the ipsilateral cerebral peduncle region in three contiguous slices in two representative participants. L, Left; R, right. ***C***, The LC was identified as spots with high signals adjacent to the fourth ventricle in three contiguous slices in the same participants. ***D–F***, Means and SEs of (***D***) the task accuracy, (***E***) SN area, and (***F***) LC CNR in patients with PD and HCs. Asterisks indicate significant group differences (*p* < 0.05). ***G***, Correlation between the total SN area and UPDRS III nontremor score. Solid line, *p* < 0.05; dotted lines, 95% confidence intervals.

Therefore, fMRI was combined with a computerized digit-ordering task (Fig. 1*A*) that highlighted the flexible manipulation of sequences by contrasting reordering and recall with pure recall trials. In PD, sequence manipulation is often impaired, although sequence maintenance is preserved (Ma et al., 2019). In pure recall trials, participants recalled a sequence of digits in the order they had been presented. In reorder and recall trials, they had to rearrange the digits in ascending order and recall the new sequence. First, we sought to replicate group differences in task accuracy (Ye et al., 2021) and SN integrity (Sasaki et al., 2006; Wang et al., 2018). Second, we wanted to detect whether the basal ganglia exhibit altered regional activation or functional connectivity with the SN. Third, we aimed to examine whether altered basal ganglia activation or functional connectivity mediates the effect of SN on task accuracy. In particular, we asked whether the ordering-related accuracy cost correlates with the basal ganglia activation or functional connectivity with the SN (brain–behavior relationship) and whether the ordering-related basal ganglia activation correlates with the SN integrity or daily dose of dopaminergic drugs (structure–function relationship).

## Materials and Methods

This study was approved by the ethics committee of the Chinese Academy of Sciences, Institute of Neuroscience, according to the Declaration of Helsinki. Each participant signed a written informed consent before participating in this study.

### 

#### 

##### Patients and clinical assessment

An a priori power analysis with G*Power 3.1 (Faul et al., 2009) suggested that a total of 58 participants (two groups) would be required to detect a small-to-medium group difference (effect size 0.3) using a repeated measures ANOVA (α = 0.05, power = 0.80). Therefore, we included 29 patients with idiopathic PD (17 women) at the Zhongshan Hospital Department of Neurology between 2019–2020. Inclusion criteria were the following: (1) PD diagnosis, according to the Movement Disorder Society clinical diagnostic criteria for PD (Postuma et al., 2015); (2) Hoehn and Yahr Scale, 1–3; (3) age 50–75 years; (4) education >6 years; and (5) right-handedness. Exclusion criteria were the following: (1) a history of other neurologic diseases (e.g., epilepsy, stroke, or brain injury); (2) treatment with benzodiazepines, neuroleptics, or antidepressants; (3) alcohol or drug abuse; (4) possible current depression [Chinese version of the Geriatric Depression Scale (GDS) >10/30]; (5) possible dementia [Chinese version of the Montreal Cognitive Assessment–Basic (MoCA–BC) <21/30); (6) low working memory spans (adaptive digit-ordering and digit span forward tests <4); or (7) contraindications to MRI. Five additional patients were measured but excluded from data analysis because of excessive head motion during scanning.

One patient was unmedicated. All other patients were assessed on their regular antiparkinsonian drugs, including levodopa (*n* = 24), pramipexole (*n* = 15), amantadine (*n* = 11), rasagiline (*n* = 8), piribedil (*n* = 6), and selegiline (*n* = 6). The levodopa equivalent daily dose was calculated (Tomlinson et al., 2010). Table 1 shows demographic, clinical, and neuropsychological data.

**Table 1.** Demographic, clinical, and neuropsychological data (means, SDs, and group differences)

Features/Measures	PD (*N* = 29)	HC (*N* = 29)	Group differences (*p* values)
Male/female	12/17	11/18	0.79
Age (years)	64.6 (5.0)	63.6 (6.1)	0.51
Education (years)	10.7 (1.9)	11.0 (2.3)	0.62
Body mass index	23.3 (3.5)	22.7 (2.8)	0.50
Motor symptoms			
Age of onset (years)	59.8 (6.2)	-	-
Disease duration (years)	4.8 (4.3)	-	-
Hoehn and Yahr stage	2.0 (0.6)	-	-
UPDRS III score	24.3 (9.3)	-	-
Levodopa equivalent daily dose			
Total (mg/day)	391.1 (214.7)	-	-
Levodopa (mg/day)	215.2 (134.4)	-	-
D_2/3_ receptor agonists (mg/day)	65.2 (50.3)	-	-
Cognition			
Montreal Cognitive Assessment Basic score	26.3 (1.3)	27.7 (1.0)	<0.001*
Digit span forward	7.6 (1.3)	7.6 (1.2)	0.92
Adaptive digit ordering	5.7 (1.6)	5.6 (1.7)	0.87
Other nonmotor functions			
Geriatric Depression Scale score (30 item)	4.6 (3.4)	3.6 (2.2)	0.17
REM Sleep Behavior Disorder Screening Questionnaire score	4.1 (3.3)	1.9 (2.2)	0.004*
Epworth Sleep Scale score	3.9 (2.9)	2.9 (2.7)	0.21

Group differences, *p* values of two-sample *t* tests or Kruskal–Wallis test as appropriate; asterisks indicate significant differences thresholded at *p* < 0.005 (Bonferroni correction for 10 tests).

##### Healthy control subjects

We included 29 healthy controls (HCs; 18 women) matched in age, education, and handedness. Exclusion criteria were the following: (1) a history of significant neurologic or psychiatric diseases, (2) alcohol or drug abuse, (3) possible current depression (GDS >10/30), (4) possible dementia or mild cognitive impairment (MoCA–BC <26/30), (5) low working memory spans (adaptive digit-ordering and digit span forward tests <4), or (6) contraindications to MRI. Six additional HCs were measured but excluded from data analysis because of excessive head motion during scanning.

##### Experimental design and procedure

All participants completed the computerized digit-ordering task (Fig. 1*A*; Ye et al., 2020), including a practice block (4 min) and two experimental blocks during scanning (7 min each). The task used a slow-event-related design, including interleaved 30 pure recall trials and 32 reorder and recall trials. In each trial, participants read four different digits and memorized them in ascending order over a short delay. In pure recall trials, the digits were presented already in ascending order. In reorder and recall trials, the digits were randomized, and participants had to reorder them. After the delay, participants were shown a pair of digits and positions. They judged whether the digit matched the position in the target order and responded by pressing the yes or no button with the right hand within 5 s.

##### Analysis of behavioral data

We controlled the quality of behavioral data by monitoring premature (reaction time shorter than 0.1 s) and inattentive responses (reaction time longer than 2.5 SDs above the mean). Only one patient made a premature response. Participants made only a few inattentive responses (∼2% in each group). The premature and inattentive responses were excluded from further analysis.

We examined whether PD patients performed less accurately (percentage of correct trials) than HCs using a repeated measures ANOVA (*p* < 0.05). The ANOVA had two factors, Group (PD, HC) and Trial Type (pure recall, reorder and recall).

##### Acquisition of neuromelanin-sensitive MRI and fMRI data

Brain imaging data were acquired on a Siemens 3T Tim Trio MRI scanner with an eight-channel head coil. High-resolution T1-weighted images used a magnetization-prepared rapid gradient-echo sequence (192 sequential sagittal slices, 2300 ms time of repetition, 3 ms time of echo, 9° flip angle, 256 × 256 mm^2^ field of view, 1 mm thickness, no gap, and 1 × 1 mm^2^ in-plane resolution). Neuromelanin-sensitive T1-weighted images used a fast spin-echo sequence (16 interleaved axial slices, 1000 ms time of repetition, 13 ms time of echo, 90° flip angle, 256 × 256 mm^2^ field of view, 2.5 mm thickness, no gap, and 0.6 × 0.5 mm^2^ in-plane resolution). Functional T2-weighted images used a standard echo-planar imaging sequence (47 interleaved ascending axial slices, 3000 ms time of repetition, 30 ms time of echo, 90° flip angle, 192 × 192 mm^2^ field of view, 3 mm thickness, no gap, and 2 × 2 mm^2^ in-plane resolution).

##### Analysis of neuromelanin-sensitive MRI data

Neuromelanin-sensitive MRI data were analyzed with Jim 8 (Xinapse Systems). We measured the SN area using a semiautomatic approach to minimize subjective bias (Ogisu et al., 2013). High T1 signals of the SN were visible in three contiguous slices in most participants (Fig. 1*B*). Two circular regions of interest (10 mm^2^ each) were manually placed in the bilateral cerebral peduncles adjacent to the SN in each slice. The SN was identified as a cluster of voxels with a signal intensity that was 2.5 SDs above the mean signal intensity of the ipsilateral cerebral peduncle region. The SN area was averaged across three slices. We also measured the SN area with 2.75 and 3 SDs and obtained similar results.

As a validity check, we also measured the LC contrast-to-noise ratio (CNR; Wang et al., 2018; Li et al., 2019). High T1 signals of the LC were visible in three contiguous slices in most participants (Fig. 1*C*). Circular regions of interest were manually placed in the bilateral LC (2 mm^2^ each) and pons (20 mm^2^) of each slice. The LC CNR was defined as the difference between the mean LC signal intensity and the mean pontine signal intensity divided by the SD of the pontine signal intensity. The LC CNR was averaged across three slices.

Two researchers measured the SN area and LC CNR independently. The measurements were highly consistent (intraclass correlation coefficient, SN area: *r* = 0.99, *p* < 0.001; LC CNR: *r* = 0.98, *p* < 0.001). We used the mean measurements for further analysis. First, we examined whether PD patients showed smaller SN areas and lower LC CNR than HCs using a repeated measures ANOVA (*p* < 0.025). The ANOVA had two factors, Group (PD, HC) and Side (left, right). Second, as a validity check, we examined whether the total SN area (sum of two sides) correlated with the severity of motor symptoms [Unified Parkinson's Disease Rating Scale (UPDRS) III score, *p* < 0.05], separating the tremor and nontremor scores (Lewis et al., 2005b). The tremor score was derived from the sum of items 20 and 21 divided by 7 (the number of included items) to assess the severity of rest, action, and postural tremor. The nontremor score was derived from the sum of items 18, 19, 22, and 27–31 divided by 12 (the number of included items) to assess speech, facial expression, rigidity, rising from a chair, posture, gait, postural stability, and body bradykinesia and hypokinesia.

##### Preprocessing and analysis of fMRI data

Functional MRI data were preprocessed using Statistical Parametric Mapping 12 (SPM12; Revision 7219, www.fil.ion.ucl.ac.uk/spm). The first three images of each block were discarded to allow magnetization equilibration. Other images were corrected for slice acquisition time difference, realigned to a mean functional image, registered to the high-resolution T1-weighted image, normalized to the Montreal Neurologic Institute (MNI) coordinate system, resampled to voxels of 2 × 2 × 2 mm^3^, smoothed with a Gaussian kernel of 4 mm full-width half-maximum, and filtered with a 128 s high-pass filter.

We controlled fMRI data preprocessing quality by monitoring the scan-to-scan total displacement (Wilke, 2012) and spatial normalization (visual inspection). PD patients did not move more than HCs in terms of total displacement (two-sample *t* test, *p* = 0.70).

First, we replicated the ordering-related regional activation. The general linear model convolved a design matrix with a canonical hemodynamic response function at the subject level. The design matrix included correct and incorrect pure recall and reorder and recall trials as separate regressors. The total displacement was included as a nuisance regressor. Each trial was time locked to its onset and modeled with its entire duration (14–16 s). Classical parameter estimation was applied with a one-lag autoregressive model. The ordering-related activation was defined as reorder and recall versus pure recall. A whole-brain two-sample *t* test was conducted at the group level (voxel level, *p* < 0.001; cluster-level, *p* < 0.05, familywise error correction).

Second, we detected group differences in the regional activation. The regions of interest were derived from an independent fMRI dataset (45 PD patients and 45 HCs; Ye et al., 2021), including the left caudate nucleus, left globus pallidus, left subthalamic nucleus, left dorsolateral prefrontal cortex (BA9), premotor cortex (BA6), left thalamus, and left SN. The percent signal change relative to the whole-brain mean signal intensity was extracted from each region and entered into a repeated measures ANOVA (*p* < 0.05). The ANOVA had three factors, Group (PD, HC), Region (seven regions), and Trial Type (pure recall, reorder and recall).

Third, we examined whether PD patients showed weaker functional connectivity between the bilateral SN and left caudate nucleus, left globus pallidus, and left subthalamic nucleus. The time courses were extracted and demeaned for each pair of regions. The Pearson correlation coefficient was calculated, normalized using Fisher's *z* transformation, and entered into a repeated measures ANOVA (*p* < 0.05). The ANOVA had three factors, Group (PD, HC), Pair (SN-caudate, SN-pallidal, SN-subthalamic), and Side (left, right).

Fourth, we identified the brain–behavior and structure–function relationships. In particular, we examined the following: (1) whether the ordering-related accuracy cost correlated with the ordering-related caudate or subthalamic activation or the functional connectivity between the SN and caudate/subthalamic nuclei (stepwise regression, *p* < 0.05); (2) in PD patients, whether the ordering-related caudate or subthalamic activation correlated with the total SN area or the levodopa equivalent daily dose (stepwise regression, *p* < 0.05); (3) in PD patients, whether the ordering-related accuracy cost correlated with the total SN area or the levodopa equivalent daily dose (stepwise regression, *p* < 0.05); and (4) whether the ordering-related caudate activation mediated the SN effect on the ordering-related accuracy cost (mediation analysis; Baron and Kenny, 1986).

##### Data availability

Raw data have been uploaded to Dryad at https://doi.org/10.5061/dryad.b8gtht7c2.

## Results

### Group differences in task accuracy

Figure 1*D* presents task accuracy of the digit-ordering task in each group. We examined group differences in accuracy using an ANOVA with two factors, Group (PD, HC) and Trial Type (pure recall, reorder and recall). Main effects of Group (*F_(_*_1,56)_ = 7.93, *p* = 0.007, η^2^ = 0.12) and Trial type were found (*F*_(1,56)_ = 3.57, *p* = 0.06, η^2^ = 0.06), but no interaction (*F* < 1). It replicated previous findings (Ye et al., 2021) that participants tended to be less accurate in reorder and recall than pure recall trials (ordering-related accuracy cost), and that accuracy of PD patients was less accurate than that of HCs.

### Group differences in substantia nigra areas

Figure 1*E* presents SN areas in each group. We examined group differences in the SN area using an ANOVA with two factors, Group (PD, HC) and Side (left, right). Main effects of Group (*F*_(1,56)_ = 9.87, *p* = 0.003, η^2^ = 0.15) and Side (*F*_(1,56)_ = 66.39, *p* < 0.001, η^2^ = 0.55), and an interaction between Group and Side were found (*F*_(1,56)_ = 14.11, *p* < 0.001, η^2^ = 0.20). The left SN was larger than the right SN in both groups. PD patients showed smaller SNs than HCs, especially on the left side. In PD patients, the total SN area (sum of two sides) correlated with the UPDRS III nontremor score (*r* = −0.42, *p* = 0.03; Fig. 1*G*). PD patients with smaller SNs showed more severe nontremor symptoms (e.g., rigidity). There was no correlation between the SN area and UPDRS III tremor score (*p* = 0.82).

### Group differences in LC contrast-to-noise ratio

Figure 1*F* presents LC CNR in each group. We examined group differences in the LC CNR using an ANOVA with two factors, Group (PD, HC) and Side (left, right). Main effects of Group (*F*_(1,56)_ = 12.81, *p* = 0.001, η^2^ = 0.19) and Side (*F*_(1,56)_ = 142.38, *p* < 0.001, η^2^ = 0.72) were found, but no interaction (*F* < 1). It replicated previous findings (Li et al., 2019) that PD showed a lower LC CNR than HC. The LC CNR was higher on the right than on the left side in both groups.

### Group differences in basal ganglia activation

**Figure 2.**
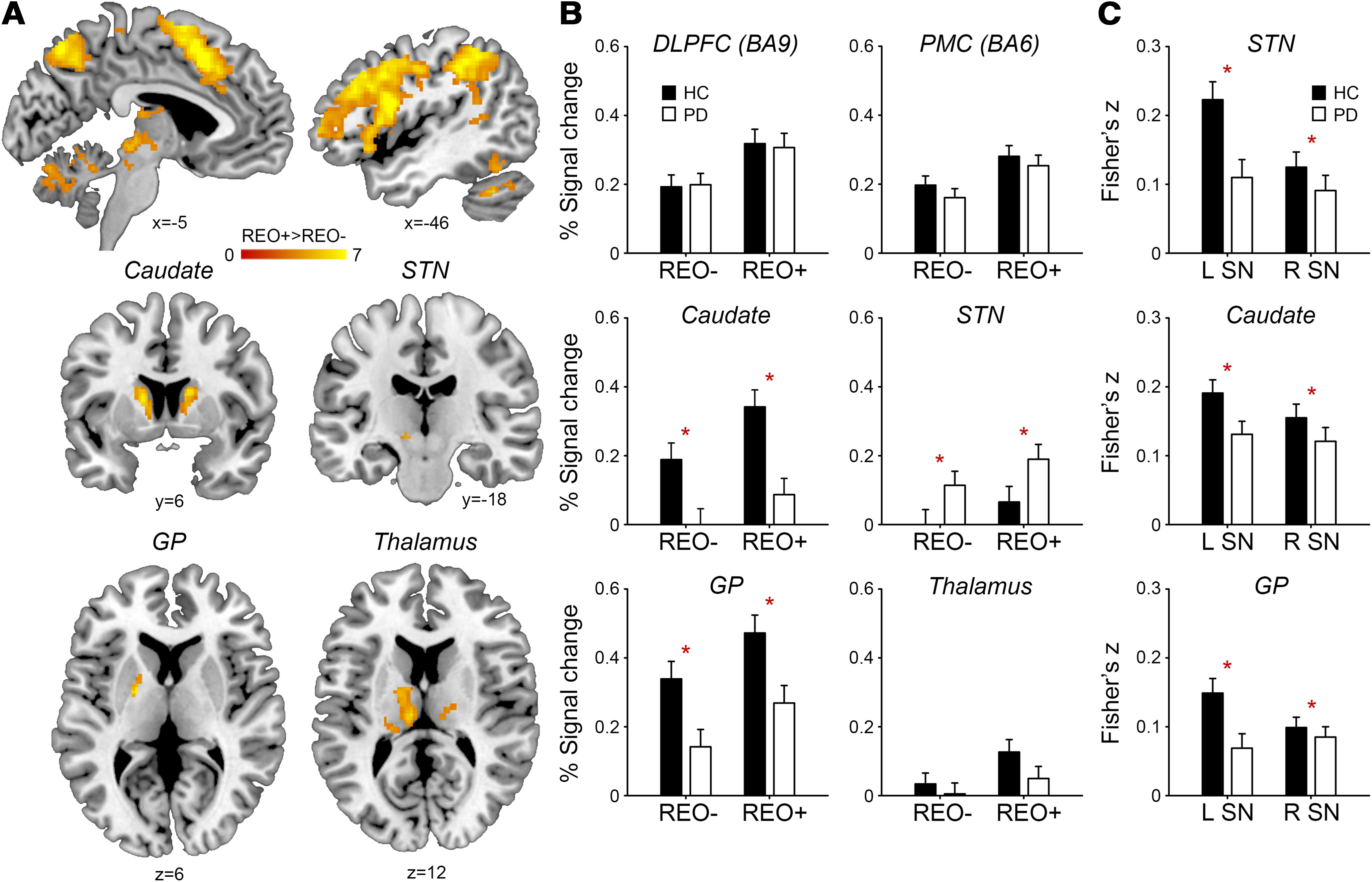
fMRI data in patients with PD and HCs. ***A***, Ordering-related regional activation across groups. STN, Subthalamic nucleus; GP, globus pallidus. The color bar indicates *t* values. Coordinates are in MNI space. ***B***, Means and SEs of the percent signal change for pure recall (REO−) and reorder and recall trials (REO+). Asterisks indicate significant group differences (*p* < 0.05). DLPFC, Dorsolateral prefrontal cortex; PMC, premotor cortex. ***C***, Means and SEs of the normalized correlation coefficients between the time courses of the SN and basal ganglia. L, Left; R, right.

First, we replicated the ordering-related regional activation across groups (Ye et al., 2020, 2021). Regional activations were greater for reorder and recall than pure recall trials (whole-brain two-sample *t* test; voxel level, *p* < 0.001; cluster level, *p* < 0.05, familywise error correction) in the dorsolateral and ventrolateral prefrontal cortex, premotor cortex, posterior parietal cortex, caudate nucleus, globus pallidus, subthalamic nucleus, and thalamus (Fig. 2*A*; Table 2).

**Table 2.** Ordering-related regional activation across groups

Regions	Brodmann area	Side	Peak in MNI (*x*, *y*, *z*)	*t* values	Cluster size (voxels)
Dorsolateral prefrontal cortex	9/46	L	[−46, 10, 36]	7.63	1124
		R	[48, 10, 32]	5.35	193
Ventrolateral prefrontal cortex	44/45	L	[−44, 8, 22]	8.97	790
		R	[56, 16, 20]	6.06	306
Premotor cortex	6	L/R	[−4, 6, 66]	9.86	2746
Posterior parietal cortex	7	L	[−8, −70, 52]	7.81	633
		R	[12, −66, 58]	6.53	271
Caudate nucleus	-	L	[−16, 0, 16]	7.28	161
		R	[14, 0, 16]	6.85	206
Globus pallidus	-	L	[−20, −4, 6]	6.65	32
Subthalamic nucleus	-	L	[−12, −18, −6]	4.55	6
Thalamus	-	L	[−16, −8, 18]	6.12	370
		R	[14, −16, 6]	6.00	106

MNI coordinate system.

Figure 2*B* presents percent signal changes in regions of interest, including the left caudate nucleus, left globus pallidus, left subthalamic nucleus, left dorsolateral prefrontal cortex (BA9), premotor cortex, and left thalamus. We examined group differences in the regional activation using a repeated measures ANOVA with three factors, Group (PD, HC), Region (seven regions), and Trial Type (pure recall, reorder and recall). Interactions between Group and Region (*F*_(6,336)_ = 6.25, *p* < 0.001, η^2^ = 0.11) and between Region and Trial type were found (*F*_(6,336)_ = 2.53, *p* = 0.05, η^2^ = 0.05), in addition to the main effects of Region (*F*_(6,336)_ = 21.22, *p* < 0.001, η^2^ = 0.30) and Trial Type (*F*_(1,56)_ = 80.75, *p* < 0.001, η^2^ = 0.62). Different regions responded differently to the task and disease.

To better understand the interaction, we conducted ANOVAs separately for each region, with two factors, Group (PD, HC) and Trial Type (pure recall, reorder and recall). In the left caudate nucleus and globus pallidus, main effects of Group (caudate: *F*_(1,56)_ = 12.24, *p* = 0.001, η^2^ = 0.19; pallidal: *F*_(1,56)_ = 8.29, *p* = 0.006, η^2^ = 0.14) and Trial type were found (caudate: *F*_(1,56)_ = 58.21, *p* < 0.001, η^2^ = 0.52; pallidal: *F*_(1,56)_ = 38.07, *p* < 0.001, η^2^ = 0.42), but no interaction (caudate, *F*_(1,56)_ = 2.76, *p* = 0.10; pallidal, *F* < 1). PD patients showed weaker caudate and pallidal activation than HCs (pairwise comparisons with Bonferroni correction: caudate, *p* = 0.001; pallidal, *p* = 0.006). In the left subthalamic nucleus, the main effect of Group (*F*_(1,56)_ = 4.56, *p* = 0.04, η^2^ = 0.08) and Trial type were found (*F*_(1,56)_ = 7.74, *p* = 0.008, η^2^ = 0.13) but no interaction (*F* < 1). However, PD patients showed greater subthalamic activation than HCs (*p* = 0.008). There was no main effect of Group in the left dorsolateral prefrontal cortex (*F* < 1), premotor cortex (*F* < 1), left thalamus (*F*_(1,56)_ = 1.26, *p* = 0.27), or left SN (*F* < 1).

### Group differences in functional connectivity between substantia nigra and basal ganglia

Figure 2*C* presents normalized correlation coefficients between the time courses of the SN and basal ganglia. We examined whether PD patients showed weaker functional connectivity between the bilateral SN and left basal ganglia using a repeated measures ANOVA with three factors, Group (PD, HC), Pair (SN-caudate, SN-pallidal, SN-subthalamic), and Side (left, right). Main effects of Group (*F*_(1,56)_ = 8.14, *p* = 0.006, η^2^ = 0.14), Pair (*F*_(2,112)_ = 9.01, *p* = 0.001, η^2^ = 0.15), and Side (*F*_(1,56)_ = 9.20, *p* = 0.004, η^2^ = 0.15), and an interaction between Group and Side were found (*F*_(1,56)_ = 4.57, *p* = 0.04, η^2^ = 0.08). The left SN was more strongly connected with the basal ganglia than the right SN (pairwise comparisons with Bonferroni correction, *p* = 0.004). The caudate and subthalamic nuclei were more strongly connected with the SN than the globus pallidus (SN-caudate vs SN-pallidal, *p* < 0.001; SN-subthalamic vs SN-pallidal, *p* = 0.04). PD patients showed weaker basal ganglia functional connectivity with the SN than HCs (*p* = 0.006), especially with the left SN.

### Brain–behavior and structure–function relationships

**Figure 3.**
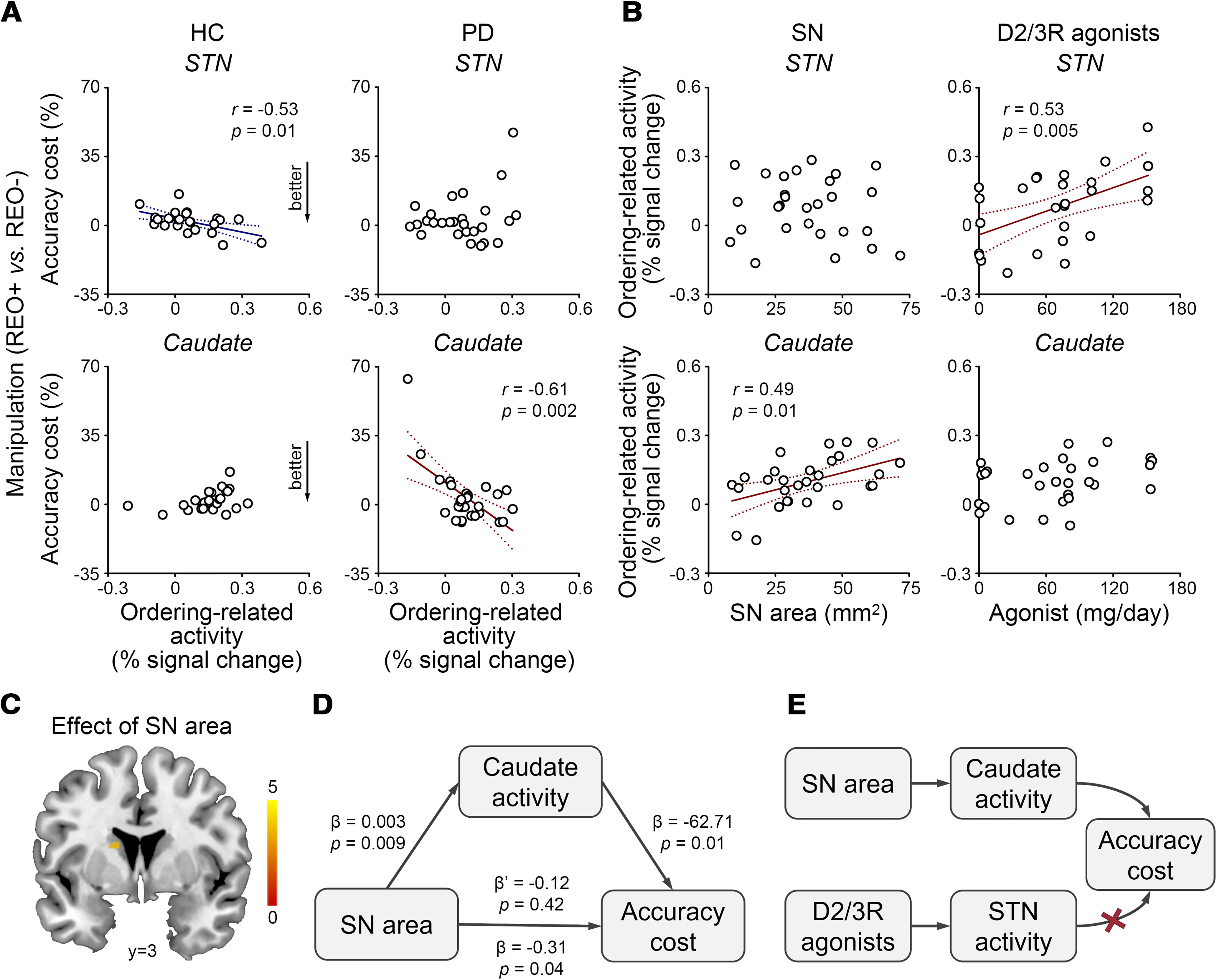
Brain–behavior and structure–function relationships in patients with PD and HCs. ***A***, In HCs, the ordering-related accuracy cost correlated with the ordering-related subthalamic activation (STN) but not the ordering-related caudate activation when the functional connectivity between the SN and caudate/subthalamic nuclei was controlled (partial correlation). In PD patients, the ordering-related accuracy cost correlated with the ordering-related caudate activation but not the ordering-related subthalamic activation when the functional connectivity between the SN and caudate/subthalamic nuclei was controlled. REO−, Pure recall; REO+, reorder and recall; solid lines, *p* < 0.05; dotted lines, 95% confidence intervals. ***B***, In PD patients, the ordering-related subthalamic activation correlated with the levodopa equivalent daily dose of D_2/3_ receptor agonists but not the total SN area. The ordering-related caudate activation correlated with the total SN area but not the levodopa equivalent daily dose of D_2/3_ receptor agonists. ***C***, The SN effect on the ordering-related caudate activation (*p* < 0.05, small volume correction). ***D***, The ordering-related caudate activation mediated the SN effect on the ordering-related accuracy cost. ***E***, A schematic diagram of brain–behavior and structure–function relationships in PD patients.

First, the ordering-related accuracy cost correlated with the ordering-related basal ganglia activation in both groups but in different manners (Fig. 3*A*). In HCs the stepwise regression model (*F*_(1,28)_ = 4.59, *p* = 0.04, *R*^2^ = 0.18) included the ordering-related subthalamic activation (*t* = −2.14, *p* = 0.04) but removed the ordering-related caudate activation (*t* = 1.94, *p* = 0.07) and total SN-caudate/subthalamic functional connectivity (*t* = −0.29, *p* = 0.77). In PD patients, the stepwise regression model (*F*_(1,28)_ = 11.40, *p* = 0.003, *R*^2^ = 0.32) included the ordering-related caudate activation (*t* = −3.38, *p* = 0.003) but removed the ordering-related subthalamic activation (*t* = 1.42, *p* = 0.17) and total SN-caudate/subthalamic functional connectivity (*t* = −0.74, *p* = 0.47). HCs with greater ordering-related subthalamic activation and PD patients with greater ordering-related caudate activation exhibited smaller accuracy costs for sequence manipulation.

Second, in PD patients, the ordering-related basal ganglia activation correlated with the SN area and the daily dose of D_2/3_ receptor agonists (Fig. 3*B*). For the ordering-related caudate activation, the stepwise regression model (*F*_(1,28)_ = 7.62, *p* = 0.01, *R*^2^ = 0.23) included the total SN area (*t* = 2.76, *p* = 0.01) but removed the levodopa equivalent daily dose of D_2/3_ receptor agonists (*t* = 1.86, *p* = 0.07). For the ordering-related subthalamic activation, the stepwise regression model (*F*_(1,28)_ = 9.62, *p* = 0.005, *R*^2^ = 0.28) included the levodopa equivalent daily dose of D_2/3_ receptor agonists (*t* = 3.10, *p* = 0.005) but removed the total SN area (*t* = −0.57, *p* = 0.58). PD patients with larger SNs exhibited greater ordering-related caudate activation, whereas those with more daily exposure to D_2/3_ receptor agonists exhibited greater ordering-related subthalamic activation.

To confirm the SN effect on the ordering-related caudate activation, we conducted an SPM12 whole-brain one-sample *t* test in PD patients with the total SN area as a covariate (voxel level, *p* < 0.001; cluster level, *p* < 0.05, small volume correction). Figure 3*C* presents the SN effect in the left caudate nucleus [peak in MNI (−16, 0, 18), *t* = 4.07, 7 voxels]. There was no significant cluster in the subthalamic nucleus or globus pallidus.

Finally, in PD, the ordering-related accuracy cost correlated with the SN area but not the daily dose of D_2/3_ receptor agonists. The stepwise regression model (*F*_(1,28)_ = 4.77, *p* = 0.04) included the total SN area (*t* = −2.19, *p* = 0.04) but removed the levodopa equivalent daily dose of D_2/3_ receptor agonists (*t* = −0.07, *p* = 0.95). Moreover, the SN effect on the ordering-related accuracy cost was fully mediated by the ordering-related caudate activation (mediation analysis; Fig. 3*D*).

### No relationship between LC contrast-to-noise ratio and basal ganglia activation

We observed no correlation between the LC CNR and basal ganglia activation in PD patients. First, we added the mean LC CNR (mean of two sides) to the stepwise regression models for ordering-related caudate and subthalamic activations. The LC CNR was removed by both models (caudate model, *t* = 0.71, *p* = 0.48; subthalamic model, *t* = −0.39, *p* = 0.70). Second, we conducted an SPM12 whole-brain one-sample *t* test in PD patients with the mean LC CNR as a covariate (voxel level, *p* < 0.001; cluster level, *p* < 0.05, small volume correction). There was no significant cluster in the basal ganglia.

## Discussion

Patients with PD may experience trouble sequencing steps in their daily lives. Here, we demonstrate that damage to the SN, rather than the LC, correlated with basal ganglia dysfunction and poor sequencing performance in PD patients. In neuromelanin-sensitive MRI, PD patients showed smaller SN areas and lower LC CNR than HCs. In the digit-ordering task with fMRI, patients' lower accuracy was accompanied by the caudate and pallidal hypoactivation, subthalamic hyperactivation, and weakened functional connectivity between the SN and all three basal ganglia regions. PD patients with smaller SNs exhibited higher ordering-related accuracy costs. This effect was fully mediated by the ordering-related caudate activation. In particular, PD patients with greater ordering-related caudate activation, but not subthalamic activation, showed lower ordering-related accuracy costs. PD patients with larger SNs showed greater ordering-related caudate activation, whereas those with more daily exposure to D2/3 receptor agonists showed greater ordering-related subthalamic activation. The SN integrity might affect behavior by boosting the task-dependent caudate activation. The D_2/3_ receptor agonists could boost the task-dependent subthalamic activation but might not change the behavior (Fig. 3*E*). We did not observe a contribution of LC in sequential working memory (Chamberlain et al., 2006).

### Cognitive mechanisms of sequential working memory

Sequential working memory might be realized by the prefrontal competitive queuing mechanism and basal ganglia gating mechanism. The competitive queuing mechanism explains how the prefrontal cortex encodes and retrieves sequential items in working memory (Averbeck et al., 2002; Ninokura et al., 2004; Berdyyeva and Olson, 2009, 2010; Kornysheva et al., 2019). The competitive queuing model is composed of a parallel planning layer, which represents the relative priority of items as the relative strength of node activations, and a competitive choice layer, which receives one-to-one inputs from the parallel planning layer and selects the item/node with the strongest activation (Hurlstone et al., 2014). A node in the parallel planning layer can be suppressed by its corresponding node in the competitive choice layer via a feedback signal.

The gating mechanism explains how the basal ganglia balance two competing processes in working memory: robust maintenance versus dynamic updating (Hazy et al., 2006; Frank et al., 2007). When currently active working memory contents need to be updated, the gate is open to allow for the processing of incoming relevant information. When maintenance demands are relatively high, the gate is closed to inhibit incoming distracting information. The basal ganglia may interact with the competitive choice layer to dynamically adjust the node activation in the parallel planning layer, for example, enhancing items/nodes to be recalled earlier and inhibiting items/nodes to be recalled later in the new sequence (i.e., open gate). This process might be promoted by the hyperdirect frontosubthalamic pathway in healthy adults and the indirect frontostriatal pathway in adults with PD. The observation of group differences in the basal ganglia activation, but not the lateral prefrontal activation, is consistent with previous findings that patients with PD are often impaired in sequence manipulation but not sequence maintenance (Werheid et al., 2002; Ma et al., 2018).

### Contribution of the substantia nigra

Damage to the SN pars compacta not only leads to a depletion of dopamine in the nigrostriatal pathway but may also cause dopamine deficiency in the nigro-subthalamic and nigro-pallidal pathways. In human brains, the SN pars compacta send dopaminergic projections to the subthalamic nucleus (François et al., 2000) and the internal and external segments of globus pallidus (Jan et al., 2000; Plantinga et al., 2016) in addition to the striatum. In PD patients, dopamine levels in the subthalamic nucleus and globus pallidus decrease remarkably, similar to that in the striatum (Hornykiewicz, 1998). Dopamine depletion might lead to the observed weakened functional connectivity between the SN and basal ganglia.

In 6-hydroxydopamine–induced rat models of PD, the firing rate of subthalamic neurons starts to increase one day after the unilateral lesion of the SN pars compacta and remains significantly higher than that in normal controls 2 weeks after the lesion, which is accompanied by irregular and bursty firing patterns (Vila et al., 2000; Breit et al., 2007). In contrast, the firing rate of pallidal neurons decreases prominently 3 weeks after the lesion of the striatum but not after the lesion of the SN pars compacta (Breit et al., 2007). Similar changes in neuronal activity might underlie the observed subthalamic hyperactivation and pallidal hypoactivation. The subthalamic hyperactivation and caudate hypoactivation might be consequences of SN damage, whereas the pallidal hypoactivation is likely a consequence of caudate hypoactivation.

### Contribution of D_2/3_ receptor agonists

This study is consistent with previous findings that dopamine plays a role in sequential working memory (Lewis et al., 2005a). However, the exact dopaminergic mechanism is an open question. In visuospatial working memory, it is proposed that D_1_ receptors mediate the robust maintenance, whereas D_2_ receptors mediate the dynamic updating (Durstewitz and Seamans, 2008; D'Esposito and Postle, 2015). However, evidence for differential roles of D_1_ versus D_2_ receptors in sequential working memory is insufficient and conflicting. For example, Cooper et al. (1992) observed improved sequencing performance in *de novo* PD patients after a 4-month monotherapy of levodopa or bromocriptine (D_2_ receptor agonist). In contrast, Dodds et al. (2009) observed improved sequencing performance in healthy adults after a single dose of 400 mg sulpiride (D_2_ receptor antagonist) versus a placebo. In addition, Gierski et al. (2007) observed an interaction between sequential working memory and a 2-month treatment of piribedil in healthy adults; after the treatment, participants with a higher capacity showed an improvement, whereas those with a lower capacity showed impairment in verbal fluency.

The inconsistent findings may be caused by heterogeneity in the receptor binding affinity of dopamine agonists (Torti et al., 2019) or clinical characteristics of patients (Ye et al., 2016). This study cannot address the question because the patients were not randomized to monotherapy of levodopa or dopamine agonist. In China, PD patients are prescribed levodopa, dopamine agonists, or levodopa plus dopamine agonists depending on their clinical presentation (e.g., age of onset, severity of motor disability, cognitive decline, depression) and financial burden. Better approaches to this question include applying monotherapy of a particular D_2/3_ receptor agonist in *de novo* patients or withdrawing medicated patients only from dopamine agonists (Claassen et al., 2015).

### Limitations and open questions

First, the globus pallidus showed hypoactivation in PD patients on medication but hyperactivation in *de novo* PD patients (Ye et al., 2021). It raises the question whether the disease or medication causes the change from hyperactivation to hypoactivation. This study cannot tease apart the effect of the disease and medication on the pallidal activation. A longitudinal study that closely monitors the change of the basal ganglia activation in newly diagnosed PD patients from the beginning of their treatment would better address this question.

Second, we had no specific hypothesis on the PD motor subtype and sequential working memory. We observed a correlation between the SN area and the severity of nontremor symptoms in PD patients. It is consistent with the literature. Earlier clinicopathological studies have shown that the akinetic-rigid subtype exhibited more neuronal loss in the SN than the tremor-dominant subtype of PD (Paulus and Jellinger, 1991; Jellinger, 1999). A recent 7 Tesla MRI study confirmed this observation, showing that smaller SN areas correlated with higher bradykinesia-rigidity scores but not tremor scores (Poston et al., 2020). As we also observed a correlation between the SN area and the ordering-related caudate activation, it raises the question of whether PD patients with less severe bradykinesia and rigidity would show greater ordering-related caudate activation and better sequencing performance. Given the small sample size, this study cannot address this question.

### Conclusion

In conclusion, we demonstrate that the SN integrity correlated with sequential working memory in patients with PD. PD patients showed smaller SNs than healthy controls. They performed less accurately than healthy controls in a digit-ordering task, accompanied by the caudate and pallidal hypoactivation, subthalamic hyperactivation, and weakened functional connectivity between the SN and all three basal ganglia regions. PD patients with smaller SNs exhibited higher ordering-related accuracy costs. This effect was fully mediated by the ordering-related caudate activation. In particular, PD patients with larger SNs showed greater ordering-related caudate activation, which was associated with lower ordering-related accuracy costs. PD patients with more daily exposure to D_2/3_ receptor agonists showed greater ordering-related subthalamic activation, which was not associated with a behavioral change. These findings suggest that damage to the SN may lead to sequential working memory deficits in PD patients, mediated by altered basal ganglia functions.
